# Identifying and Quantifying Loss Sources in Anion-Exchange Membrane Water Electrolyzers

**DOI:** 10.1021/acselectrochem.4c00156

**Published:** 2025-01-17

**Authors:** Karam Yassin, Rinat Attias, Yoed Tsur, Dario R. Dekel

**Affiliations:** † The Wolfson Department of Chemical Engineering, Technion−Israel Institute of Technology, Haifa 3200003, Israel; ‡ The Nancy & Stephen Grand Technion Energy Program (GTEP), Technion−Israel Institute of Technology, Haifa 3200003, Israel

**Keywords:** Anion-exchange membrane water electrolyzer, Dry cathode operation, Pure water, Performance analysis, Impedance Spectroscopy, Distribution Function of Relaxation Times

## Abstract

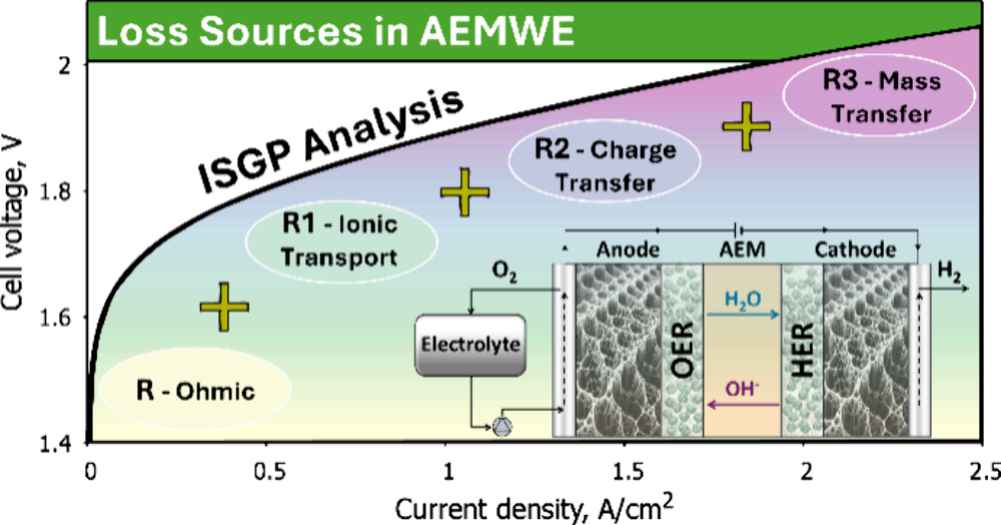

Anion-exchange membrane (AEM) water electrolyzers (AEMWEs) have gained significant attention for their ability to utilize precious-metal-free catalysts and environmentally friendly fluorine-free hydrocarbon polymeric membranes. In this study, we identify and quantify the sources of performance losses in *operando* AEMWEs using an innovative approach based on electrochemical impedance spectroscopy and MATLAB-based impedance spectroscopy genetic programming. Using this approach, we move beyond conventional equivalent circuit models to develop a proper and analytical model of the distribution function of relaxation times (DFRT), enabling a deeper analysis of Faradaic and non-Faradaic processes. We apply this framework to isolate the critical processes—ohmic, ionic transport, charge transfer, and mass transfer—across various conditions, including KOH concentration, dry cathode operation mode with different anode electrolytes (KOH, K_2_CO_3_, and pure water), cell temperature, and membrane type. Our results indicate a considerable performance reduction as the KOH concentration in the anode decreases, primarily due to the relatively high ionic transport resistance. Our observations show that the performance of dry cathode operation with KOH in the anode yields a comparable performance to dual-side electrolyte feeding due to sufficient water back-diffusion from the anode, which efficiently maintains cathode hydration. Conversely, using pure water as an electrolyte in the anode with a dry cathode significantly increases cell resistances and compromises ionic transport, underscoring the urgent need for highly conductive ionomeric materials and strategies. These insights indicate that using DFRT to evaluate the AEMWE operation by separating and associating the electrochemical phenomena could simplify system design while enabling more efficient generation of dry, pure hydrogen and advancing the technology toward commercial application.

## Introduction

The declining cost of renewable electricity has sparked a growing interest in green hydrogen as an essential factor in industrial decarbonization. This interest stems from its potential to serve as a direct fuel and a chemical feedstock, offering a pathway toward cleaner energy systems.^1−3^ Furthermore, green hydrogen holds significant promise for enhancing energy security by reducing reliance on fossil fuels and mitigating geopolitical risks associated with their supply.^4^ Water electrolysis is a key element in the green hydrogen value chain, which uses renewable energy sources to efficiently split water into hydrogen and oxygen gases.^5^

Currently, alkaline water electrolyzers (AWEs) and proton exchange membrane water electrolyzers (PEMWEs) are the commercially used technologies for producing green hydrogen.^5^ AWEs have the advantage of using non-precious metal catalysts for the electrochemical reaction, but they require highly concentrated alkaline solutions (*e.g.*, 5–7 M KOH).^6^ On the other hand, PEMWE uses a solid ion-conducting membrane. Still, the high acidity of the cell necessitates the use of expensive platinum group metal catalysts and costly fluorinate-based polymeric membranes like Nafion.^7,8^ With the beginning of the development of anion-exchange membranes (AEMs) for electrochemical energy-related devices,^9−11^ anion-exchange membrane water electrolyzers (AEMWEs) become a feasible process, able to use precious metal-free catalysts^12−15^ and solid fluorinated-free hydrocarbon-based AEMs,^16−19^ effectively merging the benefits of AWE and PEMWE technologies into a single and efficient system.^20,21^ This approach represents a promising alternative in water electrolysis for low-cost production of green hydrogen.^22^

The widespread interest in AEMWE in recent years has fueled increased activity in fundamental scientific aspects of technology.^23^ Conventionally, during the operation of the AEMWE cell, aqueous solutions such as potassium hydroxide (KOH), sodium hydroxide (NaOH), and potassium bicarbonate (K_2_CO_3_) are supplied in varying concentrations to each electrode.^5^ However, the “dry cathode” operation mode, wherein only a diluted aqueous solution or water is supplied to the anode while the cathode is not fed with any liquid or gas, is a crucial goal in advancing this technology.^20^ This strategic approach promises enhanced mass transport, facilitating efficient hydrogen release and yielding high-purity hydrogen, without further need for separation processes.^24−26^ This configuration enables the use of inexpensive stainless steel components in the balance of plant (BoP) and simplifies BoP design, substantially reducing capital expenses. Dry cathode operation also enhances hydrogen purity by eliminating the need for downstream gas separation processes, leading to reductions in both capital and operational expenses. Moreover, by pressurizing hydrogen electrochemically across the membrane, dry cathode operation could facilitate subsequent hydrogen storage, distribution, and final utilization, further reducing overall system costs and improving operational efficiency. If pure-water-fed AEMWEs can approach or match the performance of systems using KOH-based electrolytes, they could become a cost-effective and scalable solution for hydrogen production. Thus, a comprehensive analysis of cell behavior under dry cathode operation emerges as an imperative necessity. This is essential for pinpointing and mitigating performance losses, paving the way for the successful implementation of this promising approach.

In the pursuit of enhancing AEMWE cell performance, considerable attention has been directed towards the development of high-performance precious-metal-free catalysts,^27−32^ advanced AEMs and ionomers,^27,33−45^ and optimizing membrane electrode assemblies and operation conditions.^46−50^ However, despite these advancements, scant attention has been dedicated to understanding the electrochemical and transport phenomena and their contribution to resistances and losses within the cell during the device’s operation. This critical knowledge gap underscores the pressing need for meticulous elucidation of these factors, which is paramount for propelling the further advancement of AEMWE technology.

Electrochemical impedance spectroscopy (EIS) is a robust analytical technique for assessing the performance of electrochemical systems and their components occurring at the catalyst/electrolyte interface.^51^ This non-destructive method offers *in-operando* insights into performance and cell voltage losses attributed to specific components in the cell.^52^ Generally, the main contributions to losses in AEMWE arise from (a) ohmic resistance, which includes resistance to ion migration in the electrolyte, resistance to electron transport in cell components, and contact resistance; (b) electrode kinetics at the electrode/electrolyte interfaces; and (c) mass transport of reactant gases resulting collectively in the polarization resistance.^53^ Therefore, EIS measurements can help identify individual contributions to the total impedance of AEMWE cells, enabling targeted optimization of cell components and operating conditions.^54^ Typically, EIS analysis involves fitting equivalent electric circuit (EEC) models to the impedance data to extract meaningful physical insights.^55−57^ However, this approach requires prior knowledge of the system’s electrochemical behavior, and careful selection of the EEC model is crucial to prevent misinterpretation.^58^ Several studies have proposed EEC models well-suited to the impedance measurements, each with a unique EEC representing different resistive and capacitive components in the cell.^55,57,59,60^ Given these complexities, it is crucial to explore alternative methods for analyzing impedance data.^54,61^

In this study, we utilize MATLAB-based impedance spectroscopy genetic programming (ISGP) software^62^ to generate an analytical form of the distribution function of relaxation times (DFRT, a.k.a. DRT). The DFRT modeling is instrumental in accurately isolating and characterizing the different electrochemical phenomena observed in the EIS.^63−66^ This method simplifies the extraction of the effective resistance and capacitance of each electrochemical process from the DFRT data and facilitates the differentiation between Faradaic and non-Faradaic processes.^67^

Herein, we use the methodology to identify and quantify the various resistances contributing to performance losses *in-operando* AEMWEs. We first investigate the impact of KOH concentration in the electrolyte, operating cell temperature, different AEM types on the performance of AEMWEs, and feeding mode with various electrolytes. Then, using EIS and ISGP, we quantitively analyze the individual contributions of the electrochemical and transport phenomena to the overall cell resistance. Finally, we offer insights into the kinetics and discuss implications for future research directions in the AEMWE technology.

## Experimental Section

### Chemicals and Materials

A commercially available PTFE-reinforced PiperION TP-85 AEM, 15-μm thick, from W7energy (now Versogen) and AEMION AEM, 25 μm thick, from Ionomr Innovations Inc., were used to separate the anode and the cathode catalyst layers. Anodes (catalyst loading: 2.0 mg/cm^2^ of NiFe_2_O_4_, deposited on stainless steel fiber paper) and cathodes (catalyst loading: 2 mg/cm^2^ of NiFeCo, deposited on nickel fiber paper) were purchased from Dioxide Material® and used in all the AEMWE cells. Potassium hydroxide flakes AR were supplied from Bio-Lab (Israel) and were used for the electrolyte preparation for the AEMWE system and the anion conversion of the AEMs and electrodes. Potassium carbonate was supplied by Spectrum and used to prepare the electrolyte for the AEMWE system. Deionized water (18 MΩ) was obtained from a Millipore Direct-Q® 3UV system.

### AEMWE Cell Assembly

**Scheme 1:**
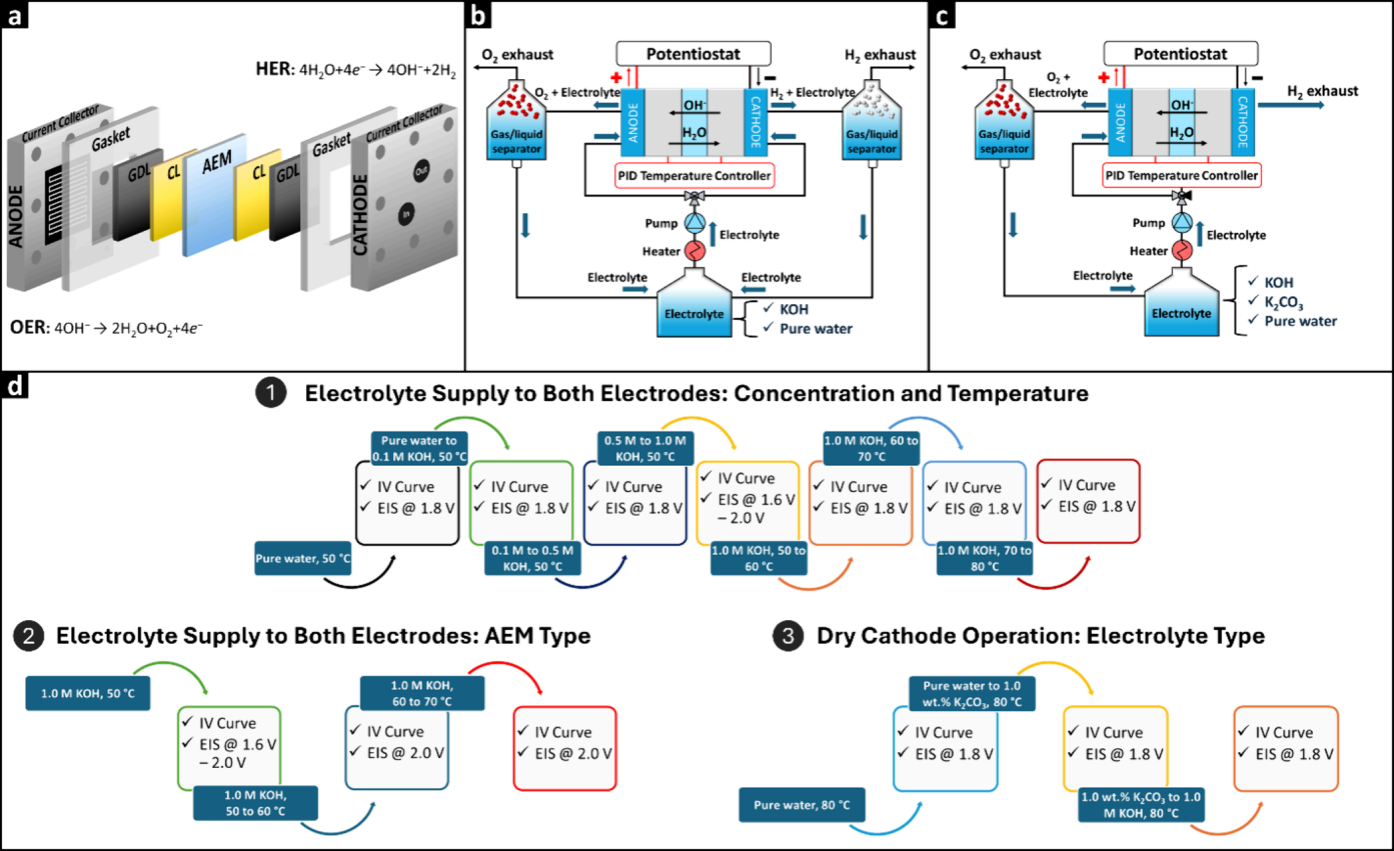
(a) Schematic Diagram of an AEMWE Cell, Lab-Scale Testing Setup for (b) Feeding KOH Electrolyte to Both the Anode and Cathode and (c) Feeding Electrolyte Only to the Anode (Dry Cathode Operation) and (d) Experimental Protocol for the Characterization of the AEMWE System under Various Conditions

Before the AEMWE tests, the membranes were ion-exchanged overnight in a 1 M KOH aqueous solution. The electrodes were immersed in 1 M KOH aqueous solution for 1 h, with solution changes occurring every 20 min. The AEMWE cells were assembled with polytetrafluoroethylene gaskets, each containing a 2.40 cm^2^ cutout in the center to ensure sufficient membrane exposure for effective electrode contact on both sides. Gas diffusion electrodes were precisely cut for the anode and cathode, with an active area of 2.25 cm^2^. The membrane electrode assembly was placed between two Nickel serpentine flow fields, each with an area of 5 cm^2^, as illustrated in Scheme 1a. Then, the cells were assembled using 3 N m of torque, ensuring a 20% compression. After assembly, the cells were immediately connected to the test station to avoid MEA drying out.

### AEMWE Cell Performance

To assess the performance of AEMWE cells, polarization curves (IV curves) and EIS measurements were carried out under varying conditions. These included two different AEMs, different electrolyte compositions such as KOH (0.1, 0.5, and 1.0 M), 1.0 wt % K_2_CO_3_ (0.07 M), and pure water at operating temperatures ranging from 50 to 80 °C. The effect of electrolyte feeding mode was also investigated comparing the performance when the electrolyte was supplied to both the anode and the cathode versus the case when the electrolyte only fed the anode (“dry cathode” mode).

A custom-built test station was used for the AEMWE cell performance test. The setup utilizes a peristaltic pump to control the feed flow of the electrolyte solution, which is split equally between the anode and cathode from a standard solution reservoir. Initially, an electrolyte solution was fed to both the anode and the cathode for a leak test (in later experiments, electrolyte solution was fed to the anode only). The cell was then heated to 50 °C using silicone heat pads controlled by a proportional-integral-derivative (PID) controller with a thermocouple while maintaining a continuous electrolyte flow. The electrolyte was pre-heated using a hot plate and maintained at 50 °C, flowing through the cell at a combined flow rate of 10 mL/min for 30 min before testing. The electrolyte was recirculated through a closed-loop system after passing through the cell (anode or both anode and cathode, depending on the experimental setup). The exit liquid streams containing gases from the two electrode compartments were separately routed to gas disengages, allowing the liquid to drop into the separate sections.

Cell performance was evaluated using a Vertex.10A potentiostat (Ivium, the Netherlands) with a current compliance of 10 A. Scheme 1b shows the setup for the case when KOH and water were supplied to both the anode and cathode. Scheme 1c illustrates the scenario where different electrolyte compositions, such as 1.0 M KOH, 1.0 wt % K_2_CO_3_ (0.07 M), and pure water, are supplied only to the anode, representing a dry cathode operating condition. Once the cell temperature stabilized, it was conditioned by stepping the voltage from 1.5 to 2.0 V in 0.1 V increments, holding each step for 5 min. The voltage was then decreased in 0.1 V increments, holding each step for 2 min until reaching 1.4 V. To ensure the cell was free of pinholes or short-circuit pathways, the cell was held at 1.0 V for further testing. The cell was subsequently returned to 1.4 V for at least 5 min to ensure stabilization. Current measurements were recorded in potentiodynamic mode (linear sweep voltammetry) across the range of 1.4–2.4 V at a scan rate of 10 mV/s mode, reaching up to 2.5 A/cm^2^. Scheme 1d overviews the sequential experimental procedure.

### Electrochemical Impedance Spectroscopy (EIS)

The EIS measurements were conducted using an Ivium potentiostat (Vertex.10A.EIS, Ivium technologies instruments), over a frequency range of 20 kHz to 100 mHz, with 20 points per decade and an alternating voltage perturbation of 10 mV, in potentiostatic mode at cell voltages ranging from 1.6 to 2.0 V, after stabilizing the cell for at least 15 min. Following acquiring the EIS, the data was transferred to ISGP for further analysis.

### Impedance Spectroscopy Genetic Programming (ISGP)

Our method uses a MATLAB-based genetic programming algorithm along with frequency dispersion transformation to analyze and extract physical information from EIS, following the approach developed by Tsur et al.^62^ The correlation between impedance and relaxation time Γ(log τ), which is a Fredholm equation of the second kind, is represented by eq 1:
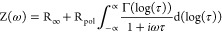
1where Z(ω) is the impedance, R_∞_ is the series resistance, R_pol_ is the total polarization resistance, Γ is the distribution function of relaxation times, τ is the relaxation time, and ω represents the frequency. Two input sets of the same impedance data or their average that differ only by white noise are utilized for validation. The Kramers–Kronig (K–K) transforms are valuable for gaining insight into the accuracy of complex impedance data, as experimental artifacts can distort it. In the ISGP procedure, the variables used are chosen based on whether the data passes the K–K transformation, a crucial step in the initial sequence.^62,68^

eq 1 represents an ill-posed inverse problem, making extracting the DFRT from the measured impedance data challenging. However, in the proper functioning of ISGP, the physically significant parameters extracted from the DFRT, such as the peak positions and their respective areas, are quite stable and can be used for further analysis with reasonable confidence. ISGP contains a library of known mathematical peaks, such as the Pseudo-delta function, Gaussian, and Lorentzian. The library also encompasses asymmetrical peaks that can be utilized in the analysis as the user needs. A linear combination of these peaks creates the distribution function. In every generation, each function creates a new sibling by adjusting the number of peaks or altering a peak type.^69,70^

The ISGP run starts with an initial population of randomly assembled models that is doubled in each generation. Each model is assessed using a “fitness function” based on the agreement of the result of eq 1 with the measured impedance data and additional features of the DFRT. The best models progress to the next generation in an iterative process. Thus, the population is evolving toward improved solutions. The evolutionary pressure prefers solutions that minimize incongruity while reducing the number of peaks and free parameters. In each generation, the DFRT with the largest merit function value is chosen, and the program stops after a predefined number of generations without change of the chosen DFRT is achieved. Ideally, each peak in the DFRT represents an electrochemical phenomenon. Since the DFRT is normalized, the effective resistance of each phenomenon is determined from the DFRT by multiplying the area under the corresponding peak by the real part of the impedance at the lowest frequency (the normalization factor). In addition, the effective capacitance related to each peak is obtained by dividing its central position in the time domain by its effective resistance.^62,66,71^

Peak number 1 (R), classified as an out-of-range pseudo-delta, is an exception. Unlike the other peaks, it does not represent a relaxation time; instead, it is used to determine the ohmic resistance accurately. Therefore, using the ISGP method allows for investigating the impact of operating conditions on each peak and quantifying resulting changes in the effective resistances and time constants in the AEMWE.

## Results and Discussion

**Figure 1:**
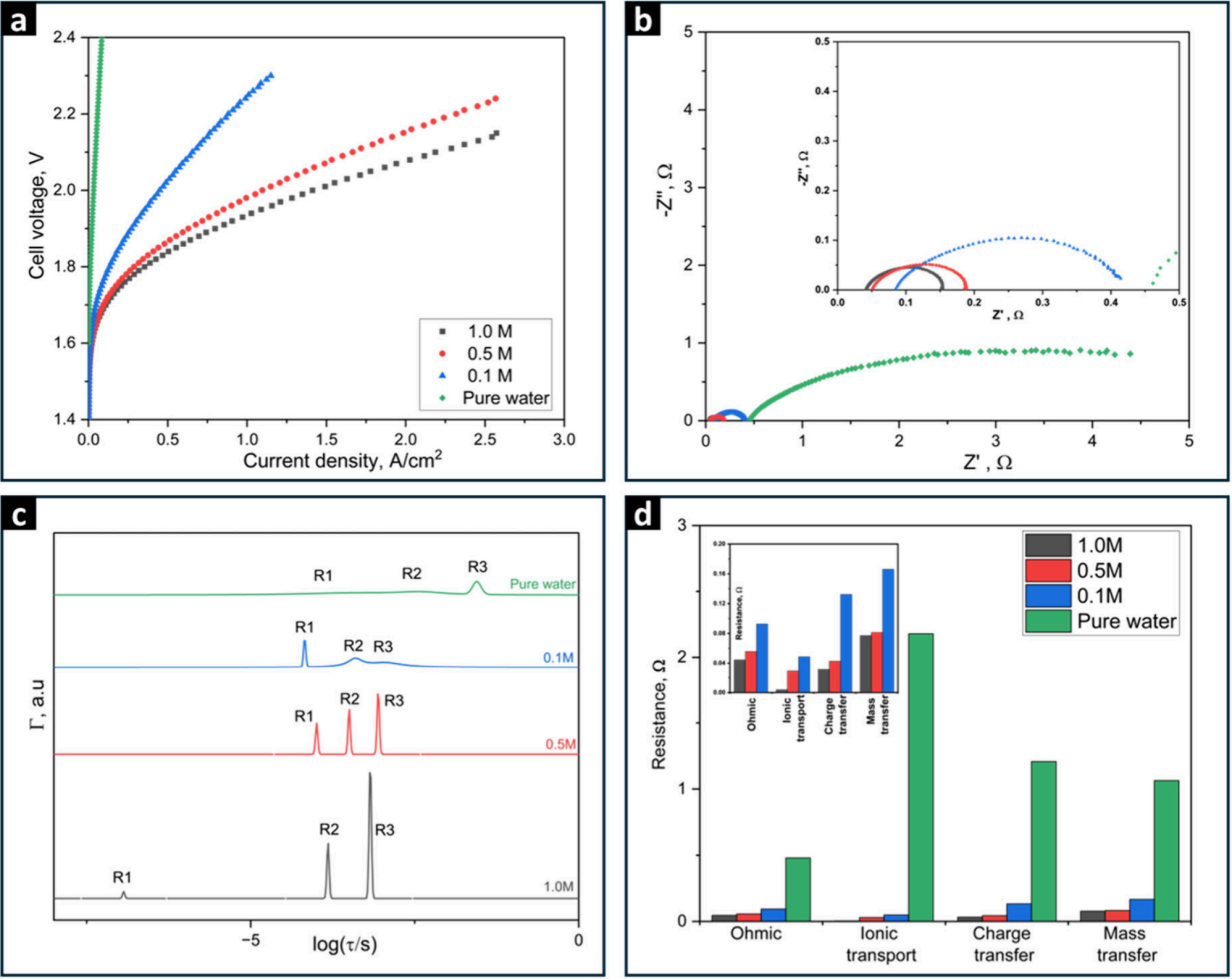
(a) Polarization curves of AEMWE cell utilizing PiperION TP-85 AEM, operated at 50 °C with different KOH concentrations (1, 0.5, 0.1, and 0.0 M (pure water)) as both anolyte and catholyte, and feed flow rates of 5 mL/min. (b) Nyquist plots of impedance spectra at 1.8 V, (c) Normalized DFRT plots, and (d) Corresponding calculated resistance values of each peak at the different electrolyte concentrations.

Fig. 1a demonstrates the impact of KOH concentration in the aqueous electrolyte, fed to both the anode and cathode (Scheme 1b), on the performance of AEMWE. The results reveal a considerable loss in overall cell performance when the concentration of KOH is reduced from 1.0 M to pure water (0.0 M KOH). For instance, the current density values measured at 2.0 V are 1.41, 1.10, 0.45, and 0.04 A/cm^2^ for the KOH concentration of 1.0, 0.5, 0.1, and 0.0 M (pure water), respectively. This represents a more than ten-fold decrease while decreasing the KOH concentration, highlighting the critical impact of the KOH on the AEMWE cell performance. Higher KOH concentration promotes sufficient ionic transport and enhances the oxygen evolution reaction kinetics, thereby reducing the overall voltage losses in the cell.^72^ This improvement is primarily attributed to enhanced hydroxide conductivity within the ionomeric materials and a higher local pH in the cell, both of which significantly decrease the ohmic resistance and the kinetic losses. This, in turn, underscores the challenges of operating AEMWE systems with pure water instead of KOH electrolytes.^20^

To elucidate the observed performance variations and underlying mechanisms, we employ EIS to assess the specific contributions to voltage losses and gain deeper insights into the impedance characteristics of the AEMWE cells. We first performed EIS at various cell voltages ranging from 1.6 to 2.0 V using 1.0 M KOH concentration, as shown in Fig. S1 and Table S1, along with the impedance spectroscopy analysis using the ISGP method. The ISGP analysis in Fig. S1 reveals four phenomena that contribute to the total resistance of the cell, hiding in the Nyquist plot of each measurement. These four distinct resistances include ohmic resistance (R), ionic transport resistance (R1), charge transfer resistance (R2), and mass transfer resistance (R3). Ohmic resistance is excluded from the DFRT, as it is categorized as an out-of-range peak and does not have a well-defined relaxation time. The analysis of Fig. S1 suggests that R1 does not represent a Faradaic phenomenon, as the resistance does not markedly change with voltage. The alteration in the time constant is due to the generation of more OH^–^ ions in the reaction, resulting in a faster phenomenon. However, the correlation between the voltage changes and the R2 and R3 values strongly suggests that the latter are Faradaic phenomena, given the substantial decrease in resistance and time constant with increasing voltage.

Fig. 1b represents the Nyquist plot of impedance spectra measured at 1.8 V for different KOH concentrations. Visually, the semi-circle is ornately decreased with increasing KOH concentration, interpreted as a loss reduction. However, EIS analysis is required to understand the influence of the electrochemical phenomena in the AEMWE cell. Fig. 1c shows the DFRT results obtained by ISGP. The ohmic resistance R is calculated to ascertain the losses within the cell during the device’s operation. According to Fig. 1d, which displays the effective resistance of each component, and Table S2, R decreases from 92.7 to 55.2 and 44.6 mΩ with increasing KOH concentration. This reduction is attributed to the enhanced OH^–^ conductivity within the ionomeric materials of the MEA.

Peak 2, defined as R1, shows a decrease in both the effective resistance and the time constant with the increase in KOH concentration, as shown in Fig. 1c and d. As the concentration increases, more OH^–^ ions can quickly reach the electrode/membrane interface, enhancing the conductivity of the membrane and resulting in a smaller peak of R1. The resistance varies from 48.7 to 4.3 mΩ and 3.8 mΩ for 0.1, 0.5, and 1.0 M KOH, respectively. This pronounced reduction in resistance between 0.1 and 0.5 M KOH underscores the substantial impact of hydroxide ion concentration on ionic transport in the cell. For pure water, the resistance to ionic transport (2.18 Ω) is higher in two orders of magnitude from 0.1 M KOH, supporting the association of R1 as an ionic transport phenomenon.

Peaks 3 and 4 in the data represent the charge transfer resistance (R2) and the mass transfer resistance (R3), respectively. The charge transfer resistance occurs at the electrode/electrolyte interfaces of the anode and cathode, while mass transfer resistance relates to the movement of reactant gases and intermediate products within the reaction.^64,67,70^ According to Fig. 1c and d, it is evident that the effective resistance of R2 and R3 decreases with the increase in KOH concentration. However, the time constants decrease moderately, supporting the association of R2 and R3 as Faradaic phenomena. The effective resistance values can also be seen in Table S2.

**Figure 2:**
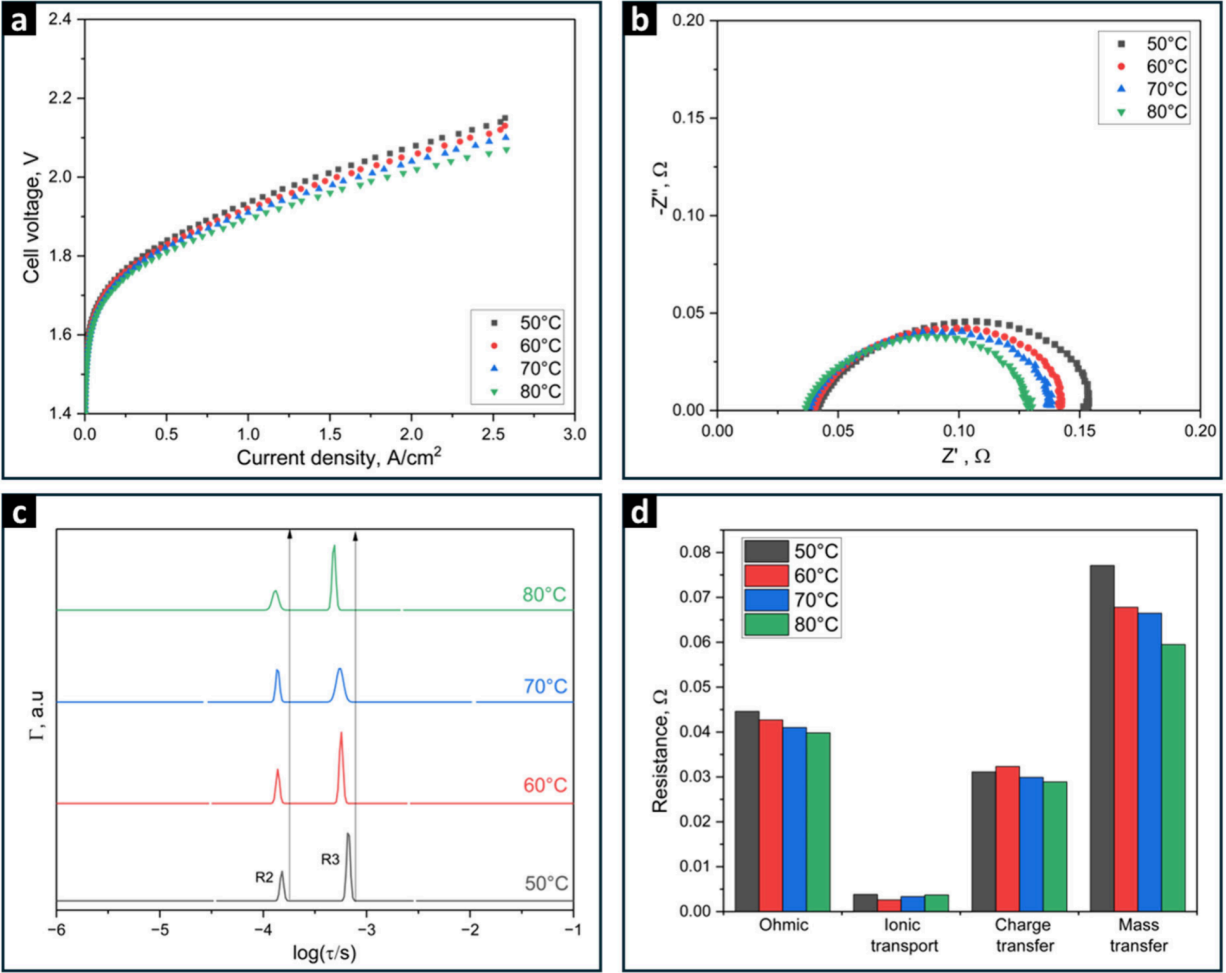
(a) Polarization curves of AEMWE cells utilizing PiperION TP-85 AEM, operated at different temperatures (50 °C–80 °C) with 1 M KOH as both anolyte and catholyte, and feed flow rates of 5 mL/min. (b) Nyquist plots of impedance spectra at 1.8 V, (c) Normalized DFRT plots, and (d) Corresponding calculated resistance values of each peak at the different operating temperatures.

Operating temperature emerges as a critical factor influencing AEMWE cell performance and the individual losses in the cell. Fig. 2a illustrates the impact of operating cell temperature on the performance of the AEMWEs fed with 1.0 M KOH solution to both anode and cathode (Scheme 1b). As anticipated, increasing cell temperature leads to improved cell performance. For instance, the current density values measured at a cell voltage of 2.0 V, increase from 1.41 to 1.85 A/cm^2^ as the temperature rises from 50 to 80 °C. This enhancement is attributed to the enhanced hydroxide conductivity through the membrane and the faster kinetics of the hydrogen evolution reaction (HER) and oxygen evolution reaction (OER) at higher temperatures.

The Nyquist plot in Fig. 2b indicates that overall resistance decreases as the temperature rises. Fig. 2c shows that the time constants for R2, the charge transfer resistance, and R3, the mass transfer resistance, are not significantly affected by the temperature and stay around -3.8 and -3.2, respectively, as shown in Table S3. Fig. 2d shows that the ohmic resistance R, charge transfer resistance R2, and mass transfer resistance R3 display a clear decreasing trend in response to temperature, similar to Arrhenius’s behavior,^73^ while the resistance to ionic transport R1 remains relatively constant, except at 50 °C. This can be attributed to the relatively small contribution of R1 to the overall cell resistance, which makes temperature dependence less pronounced. Based on this, it can be concluded that R1 is impervious to temperature changes and maintains a consistent resistance and time constant.

We next investigate the performance of AEMWE cells utilizing the AEMION membrane at different operating temperatures, with 1.0 M KOH solution supplied to both the anode and the cathode (Scheme 1b). We first performed EIS at various cell voltages ranging from 1.5 to 2.0 V using 1.0 M KOH concentration, as shown in Fig. S2, accompanied by the impedance spectroscopy analysis using the ISGP method. The ISGP analysis in Fig. S1 reveals four phenomena that contribute to the total resistance of the cell, hiding in the Nyquist plot of each measurement. This approach eliminates the need for prior system knowledge or EEC model fitting, even with variations in EIS behavior compared to the PiperION AEM system.

**Figure 3:**
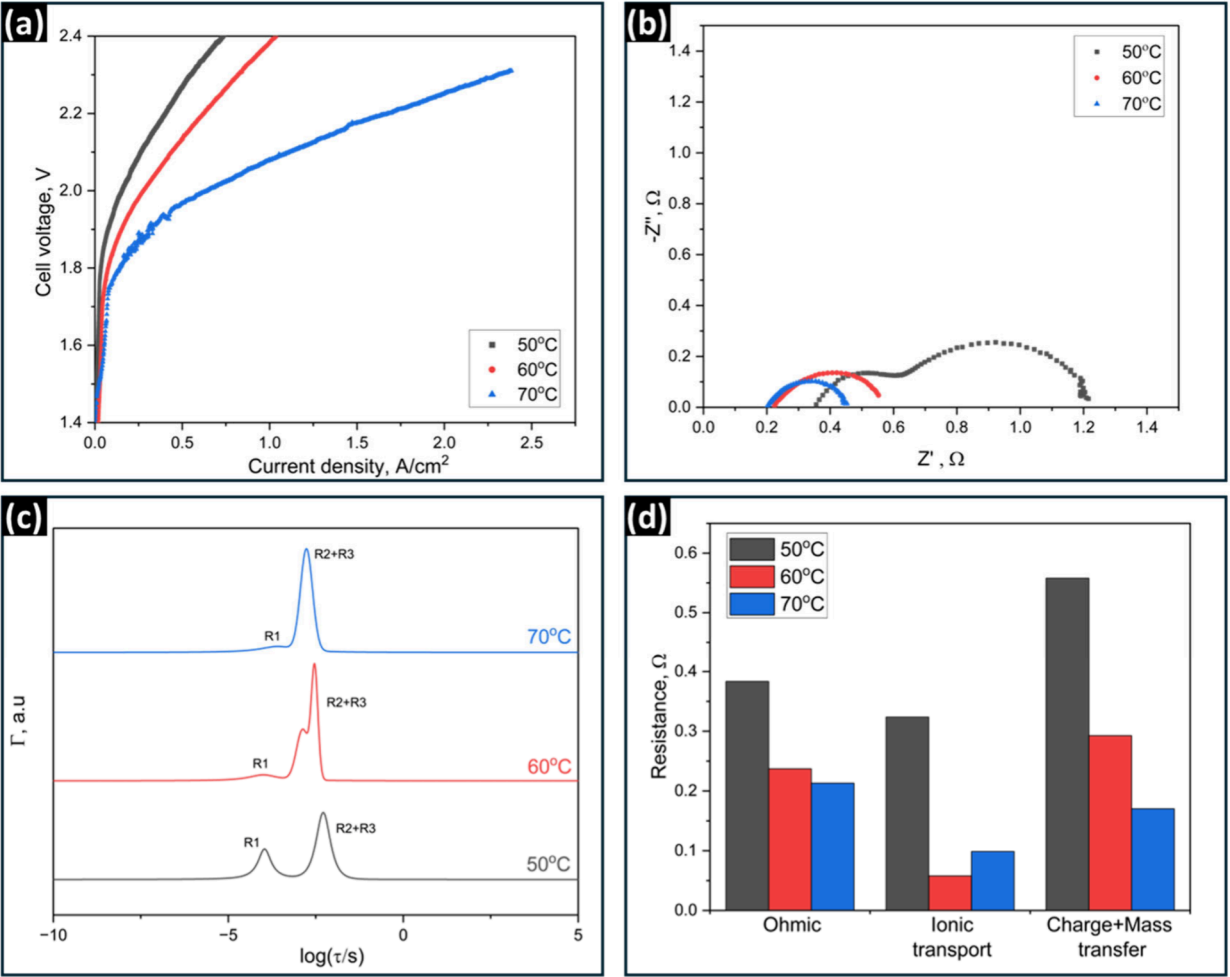
(a) Polarization curves of AEMWE cells utilizing AEMION AEM, operated at different temperatures (50 °C–70 °C) with 1 M KOH as both anolyte and catholyte, and feed flow rates of 5 mL/min. (b) Nyquist plots of impedance spectra at 2.0 V, (c) Normalized DFRT plots, and (d) Corresponding calculated resistance values of each peak at the different operating temperatures.

As expected, Fig. 3a shows a noticeable enhancement in cell performance as the temperature increases from 50 to 70° C, attributed to enhanced ionic transport and reaction kinetics. For example, the current density measured at 2.0 V increases from 0.15 A/cm^2^ at 50 °C to 0.28 A/cm^2^ at 60 °C and 0.64 A/cm^2^ at 70 °C. In comparison, when evaluating the AEMWE performance with PiperION membrane at 50 °C, we observe a striking ninefold reduction in cell performance, dropping from 1.41 to 0.15 A/cm^2^. To better understand the differences between membranes, we look at the impedance analysis of the cell.

The Nyquist plots were recorded at 2.0 V at temperatures of 50, 60, and 70 °C, and are displayed in Fig. 3b. The Nyquist plots reveal distinctions in shape compared to those of the PiperION membrane, which are attributed to the lower ionic conductivity and greater thickness of the AEMION membrane. At lower temperatures, the Nyquist plot for AEMION displays a pronounced low-frequency arc, highlighting the combined effects of charge transfer and mass transfer resistances. As the temperature increases, this arc becomes less prominent, reflecting enhanced ionic conductivity and improved water transport within the membrane. Unlike the case of the PiperION cell, the DFRT in Fig. 3c shows that R2 and R3 occur at the same time constant, making it impossible to separate the phenomena due to the overlap between the peaks. The resistance values of R2 and R3 were calculated together and are displayed in Fig. 3d. The effective resistances of charge and mass transfer at 50, 60, and 70 °C are 0.558, 0.292, and 0.170 Ω, respectively. The difficulty in separating the phenomena may be attributed to the faster occurrence of the forms of intermediates reaching the surface through the membrane.

The ohmic resistance R follows the same pattern, with values of 0.383, 0.237, and 0.213 Ω at 50, 60, and 70 °C, respectively. The decrease in charge and mass transfer resistance is primarily due to the increased mobility of ions within the membrane at higher temperatures. The OH^–^ ions are more mobile at higher temperatures due to their loosely packed state, allowing them to move more quickly between the membrane and the catalyst layer. Conversely, at lower temperatures, the movement of the OH^–^ ions is slower due to a lower diffusion coefficient, resulting in a lower availability of ions for the redox reaction, leading to higher charge transfer resistance. The ionic transport R1 represents resistance values of 0.324, 0.058, and 0.099 Ω at 50, 60, and 70 °C, respectively. Also, in this membrane, the time constant of R1 is not influenced by temperature and remains stable. Overall, compared to the PiperION membrane, the AEMION membrane displays a tenfold increase in total resistance, which can be attributed to its greater thickness and lower ionic conductivity. It must be noted that we have used the same electrodes in the membrane electrode assemblies, and not all membranes are compatible with the same electrodes. Different AEMs may need different electrodes to maximize their performance.

The final analysis examines how different electrolyte feeding modes–feeding electrolyte only to the anode (dry cathode, Scheme 1c) and feeding electrolyte to both the anode and cathode–as well as different electrolyte solutions (KOH, K_2_CO_3_, and water), affect the performance of AEMWE cells. We begin by comparing the performance of AEMWE cells with 1.0 M KOH fed only to the anode versus 1.0 M KOH fed to both the anode and cathode. Next, we evaluate the performance of the cells with a dry cathode using 1.0 M KOH, 1.0 wt % K_2_CO_3_, and pure water fed only to the anode.

**Figure 4:**
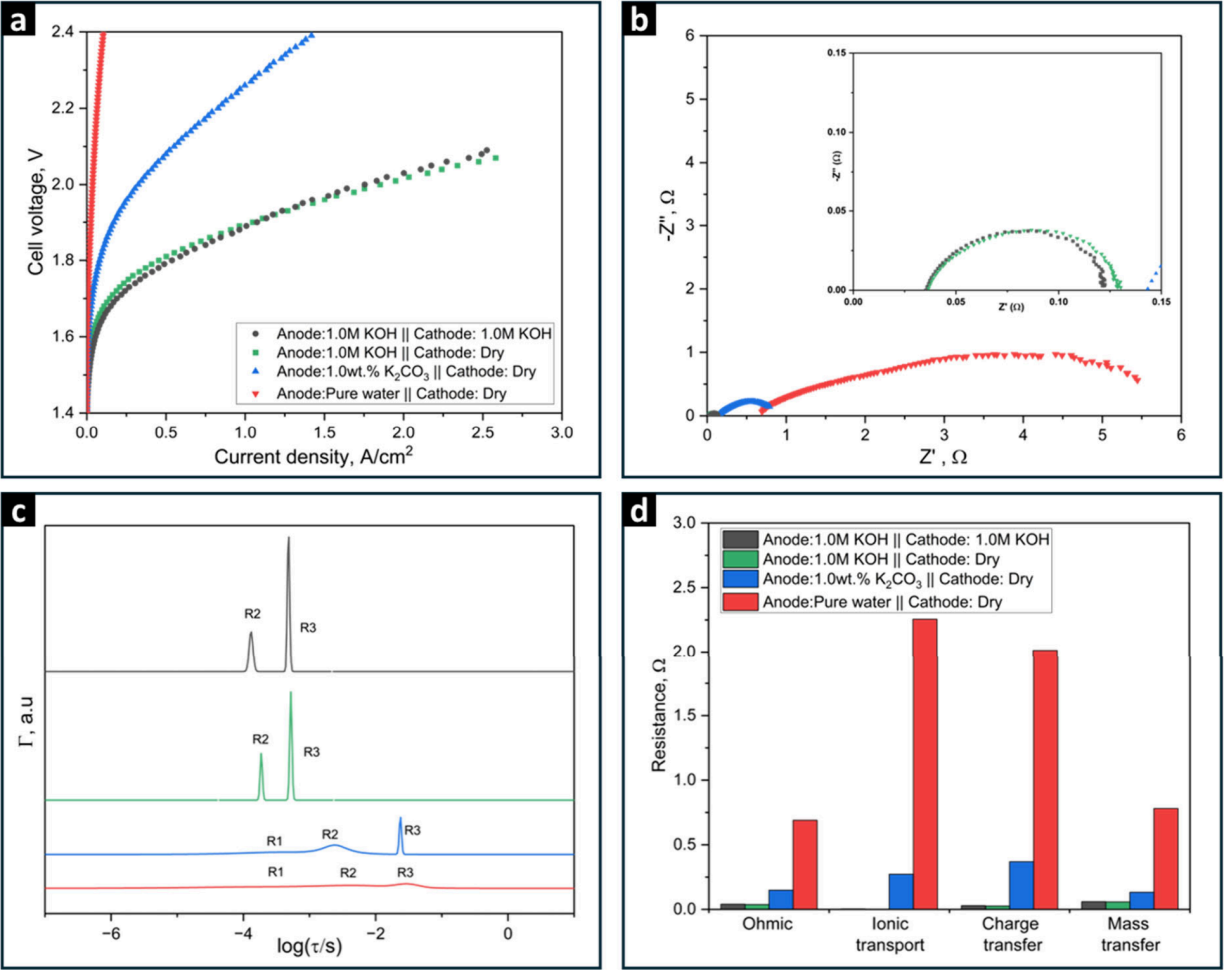
(a) Polarization curves of AEMWE cell utilizing PiperION TP-85 AEM, operated at 80 °C at different electrolyte feeding modes with electrolyte solutions and feed flow rates of 5 mL/min. (b) Nyquist plots of impedance spectra at 1.8 V, (c) Normalized DFRT plots, and (d) Corresponding calculated resistance values of each peak.

The results in Fig. 4a, reveal that the two electrolyte feeding modes exhibit fairly similar cell performance. One of the main challenges in AEMWE is cathode dehydration. Given the similarity between AEMWE and AEMFC systems, reducing AEM thickness or enhancing water back diffusion from the anode to the cathode could help mitigate this issue, maintaining a higher hydration level in the cathode.^24,74,75^ These results, though counterintuitive, suggest that water back-diffusion from the anode to the cathode may be a more effective mechanism for maintaining cathode hydration than direct electrolyte feed.^76^ Additionally, with a dry cathode, the produced hydrogen in the HER can be easily released from the cell, reducing mass transport resistance.

Next, we examine the effect of electrolyte type under dry cathode operation. The results show a significant decrease in overall cell performance as the anode electrolyte changes from a 1.0 M KOH solution to a 1.0 wt % K_2_CO_3_ (equivalent to 0.07 M KOH) solution, and further to pure water. This performance trend can be primarily attributed to ionic transport and ion type differences. KOH, with a higher concentration of hydroxide ions (1.0 M), enables more effective ion transport and lower mass transport resistance, resulting in a better overall performance. By contrast, 1.0 wt % K_2_CO_3_ (0.07 M) provides a lower concentration of conductive ions, leading to reduced catalyst utilization as carbonate ions are less effective for the OER compared to hydroxide ions. This is consistent with previous findings that switching from 1.0 M KOH to 1.0 wt % K_2_CO_3_ results in only a 1.5-fold increase in catalyst utilization, compared to a 5-fold increase with 1.0 M KOH, driven by enhanced hydroxide ion transport, an expanded catalyst-electrolyte interface, and improved reaction kinetics.^77−79^ Additionally, the presence of carbonate ions can result in membrane carbonation,^80−82^ further reducing membrane ionic transport resistance and increasing ohmic resistance. The accumulation of carbonate ions also leads to a pH gradient across the system, which raises the Nernstian voltage for electrolysis, further hindering performance.^83^

Despite these drawbacks, AEMWE with K_2_CO_3_ electrolyte offers significantly better cell performance than pure water. Although less efficient than KOH, it provides some level of ionic transport and stabilizes the pH to maintain a regulated mild alkaline environment, which can support the electrochemical OER at the anode. In contrast, pure water exacerbates these issues, resulting in severe voltage losses and a substantial decrease in electrolysis efficiency.^79^

Fig. 4b presents the Nyquist plots at 1.8 V for the four cases described above, as displayed in Fig. 4a. The results indicate that, under dry cathode operation, the AEMWE cell operating with pure water exhibits the largest semi-circle, followed by the cell operating with 1.0 wt % K_2_CO_3_, and then the cell operating with 1.0 M KOH, which displays the smallest semi-circle. Importantly, similar semi-circle characteristics are observed when operating the AEMWE with 1.0 M KOH, regardless of whether the KOH is fed solely to the anode or to both the anode and the cathode, as shown in the zoom-in plot in Fig. 4b. Fig. 4c presents the analysis of the EIS results as DFRTs. It agrees with the classification of electrochemical phenomena we performed for the cell system. In the context of ionic transport resistance, R1 exhibits a very low time constant of -7.24 for both dry cathode operation and when both the anode and cathode are supplied with 1.0 M KOH. However, when the anode contains 1.0 wt % K_2_CO_3_ and pure water, the log of time constants are -3.50 and -3.81, respectively, indicating a slower process that adds significant resistance to the cell, as depicted in Fig. 4d. Additionally, when pure water is fed to the anode, the highest effective resistance is attributed to reduced ionic transport resistance, emphasizing the need for highly conducting ionomeric materials. Conversely, when 1.0 M KOH is in the anode and the cathode is dry, R1 does not appear in the analyzed Nyquist plot by ISGP, suggesting that ionic transport in the anode is negligible with a minimal resistance of 0.37 mΩ when 0.1 M KOH is present in both the anode and the cathode. Additionally, R2 and R3 represent charge transfer and mass transfer resistance, showing high effective resistance compared to R2 and R3 for 1.0 wt % K_2_CO_3_ and pure water, as shown in Table S4. It should be noted that the relaxation time of R3 is significantly influenced by the transition from KOH to pure water as the electrolyte. The reduced ionic conductivity of pure water slows reaction kinetics, which increases the relaxation time of R3 and consequently affects the mass transfer of H_2_ and O_2_. Similar trends are observed in Fig. 1, confirming that changes in electrolyte conductivity impact both reaction kinetics and mass transfer across the electrodes. In Contrast, all resistances (R, R1, R2 and R3) are significantly lower and roughly equal for anodes with KOH solution, whether the cathode is dry or fed with 1.0 M KOH, indicating faster reaction kinetics and improved mass transport.

In summary, this study successfully identifies and quantifies the sources of performance losses *in*-*operando* AEMWEs through an innovative approach, moving beyond conventional equivalent electric circuits at different operating parameters and operation modes. The findings represent a significant step forward in understanding and optimizing AEMWE technology. Future investigation into the sources of losses during longevity tests of AEMWE systems will be essential for achieving durable AEMWE cells, for practical applications in hydrogen production.

## Conclusion

This study comprehensively evaluates the performance of AEMWEs, highlighting the role of operational parameters, including cell temperature, electrolyte composition, and feeding mode. Through an innovative EIS and ISGP approach, we successfully identify and quantify the sources of performance losses in AEMWE, revealing four dominant resistances: ohmic, ionic transport, charge transfer, and mass transfer resistances. Our findings show a significant decline in AEMWE cell performance when transitioning from 1.0 M KOH to pure water, mainly due to the increased ionic transport resistance, with pure water exhibiting resistance over two orders of magnitude higher than 1.0 M KOH. Increasing operating temperature enhances AEMWE cell performance by reducing ohmic, charge transfer, and mass transfer resistances, following an Arrhenius-like trend. Furthermore, we observe that dry cathode operation, with KOH in the anode, can maintain cathode hydration effectively through water back-diffusion, delivering comparable performance to an AEMWE with KOH supplied to both sides. Conversely, using pure water as an electrolyte in the anode with a dry cathode significantly increases cell resistances and, most critically, compromises ionic transport. These results underscore the urgent need for utilizing highly conductive materials in pure water systems and exploring additional strategies, such as operating the system at higher temperatures, which can enhance overall cell performance. Continued exploration of dry cathode operation and water management with pure water fed to the anode by identifying and quantifying loss sources within the cell is essential for enhancing AEMWE performance and operational simplicity.

## Supporting Information Available

Additional experimental results and model analysis, Figures S1–S2, Tables S1–S4 (PDF)
