# Supplementary material for: Identifying and Quantifying Loss Sources in Anion-Exchange Membrane Water Electrolyzers
Source: ACS Electrochem. 2025 Jan 17;1(5):655–66. doi: 10.1021/acselectrochem.4c00156 (PMC12051194; doi:10.1021/acselectrochem.4c00156)
Supplement: Supplementary file 1 — ec4c00156_si_001.pdf [file ec4c00156_si_001.pdf]

Supporting Information

Identifying and Quantifying Loss Sources in Anion-Exchange Membrane Water Electrolyzers

Karam Yassin [a,b‡], *Rinat Attias* [b‡], *Yoed Tsur* [a,b*] ,*and Dario R. Dekel* [a,b*]

[a] The Wolfson Department of Chemical Engineering, Technion – Israel Institute of Technology, Haifa 3200003, Israel

[b] The Nancy & Stephen Grand Technion Energy Program (GTEP), Technion – Israel Institute of Technology, Haifa 3200003, Israel

‡ These authors contributed equally to this work

* Corresponding authors

E-mail addresses: dario@technion.ac.il (D.R. Dekel), tsur@technion.ac.il (Y. Tsur)

Table of Contents

Figure S1. *(a) The polarization curve of an AEMWE cell utilizing PiperION TP-85 AEM, operated at 50°C with 1 M KOH as both anolyte and catholyte and feed flow rates of 5 mL/min. (b) Nyquist plots, (c) Normalized DFRT plots, and (d) Corresponding calculated effective resistance values of each peak at the different cell voltages ranging between 1.6-2.0 V.* S2

Table S1. *The effect of varying voltage on the resistances and time constants of the electrochemical phenomena occurring in the AEMWE cell.* S3

Table S2. *The effect of KOH concentration on the resistance and time constant of the electrochemical phenomena occurring in the AEMWE cell at 1.8V in temperature of 50°C.* S4

Table S3. *The effect of operating temperature on the resistances and time constants of the electrochemical phenomena occurring in the AEMWE cell at 1.8 V.* S4

Table S4. *The effect of operating modes on the resistances and time constants of the electrochemical phenomena occurring in the AEMWE cell at 1.8 V and temperature of 80 °C.* .S5

Figure S2. *(a) The polarization curve of an AEMWE cell utilizing AEMION AEM operated at 50°C with 1 M KOH as both anolyte and catholyte and feed flow rates of 5 mL/min. (b) Nyquist plots, (c) Normalized DFRT plots, and (d) Corresponding calculated effective resistance values of each peak at the different cell voltages ranging between 1.5-2.0 V.* S6

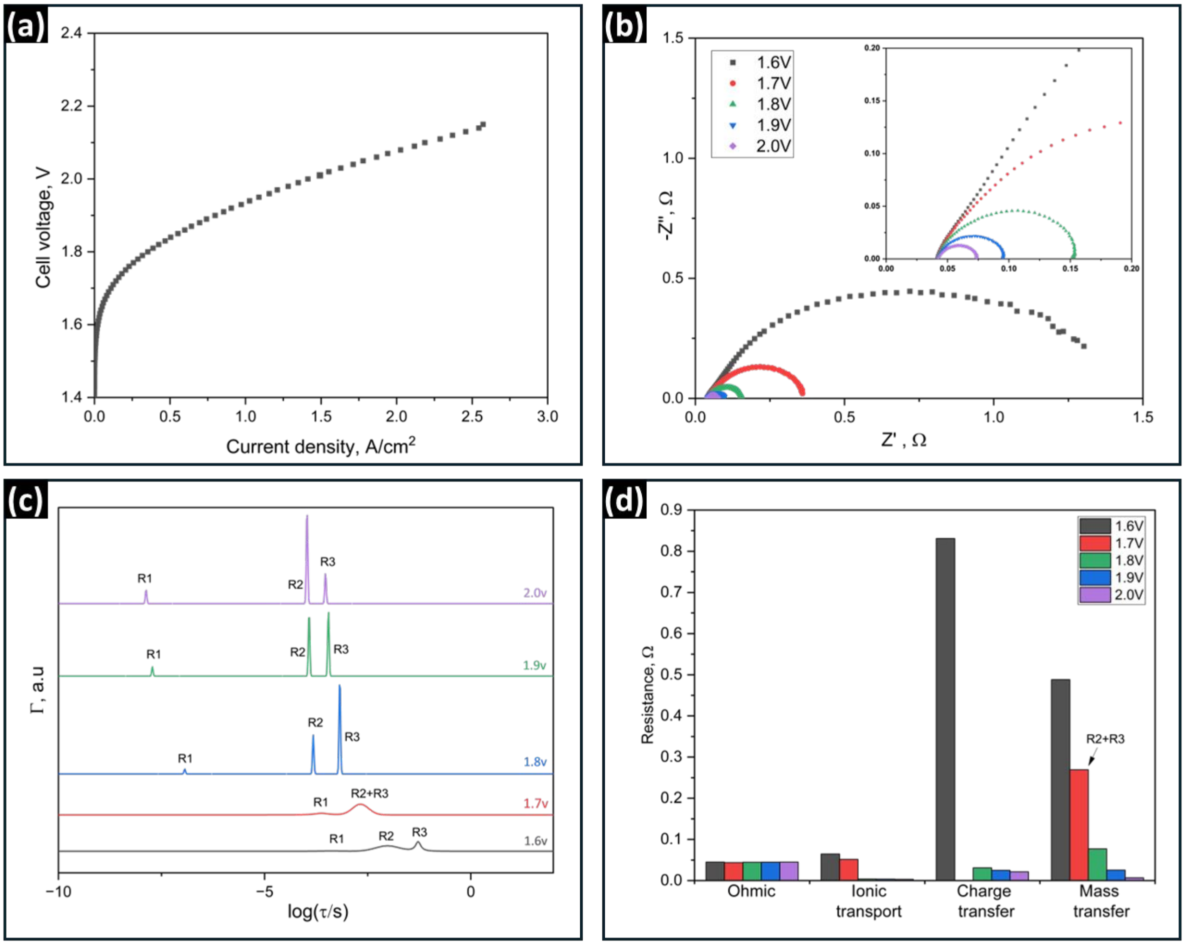

Figure S1. *(a) The polarization curve of an AEMWE cell utilizing PiperION TP-85 AEM, operated at 50°C with 1 M KOH as both anolyte and catholyte and feed flow rates of 5 mL/min. (b) Nyquist plots, (c) Normalized DFRT plots, and (d) Corresponding calculated effective resistance values of each peak at the different cell voltages ranging between 1.6-2.0 V.*

Fig. S1 shows that with increasing voltage, the time constant of all electrochemical phenomena in the range gets shorter. This means the phenomena become faster, including R1 representing the non-Faradic resistance associated with the ionic transport phenomenon. R2 and R3 represent the Faradaic phenomena due to the dramatic decrease in resistance as the voltage increases. Moreover, starting from a voltage of 1.8 V, where the impedance profile and ISGP results are similar, it is evident that the resistance to mass transfer is initially more significant than the resistance to charge transfer. However, a change in trend occurs where the resistance to charge

transfer becomes more prominent than the mass transfer limitation. According to Table S1, Ohmic resistance remains constant. However, ionic conductivity resistance decreases as the potential increases. Additionally, resistance to charge transfer (CT) and mass transfer (MT) decreases with increasing potential, except for a deviation at a voltage of 1.7 V.

Table S1. *The effect of varying voltage on the resistances and time constants of the electrochemical phenomena occurring in the AEMWE cell.*

<table>
<tr><th>Potential</th><th colspan="2">1.6 V</th><th colspan="2">1.7 V</th><th colspan="2">1.8 V</th><th colspan="2">1.9 V</th><th colspan="2">2.0 V</th></tr>
<tr><th>Peak</th><th>R [Ω]</th><th>log (τ)</th><th>R [Ω]</th><th>log (τ)</th><th>R [Ω]</th><th>log (τ)</th><th>R [Ω]</th><th>log (τ)</th><th>R [Ω]</th><th>log (τ)</th></tr>
<tr><td>Ohmic resistance</td><td>0.0450</td><td></td><td>0.0439</td><td></td><td>0.0446</td><td></td><td>0.0448</td><td></td><td>0.0451</td><td></td></tr>
<tr><td>Ionic transport</td><td>0.0646</td><td>-3.38</td><td>0.0517</td><td>-3.62</td><td>0.0038</td><td>-6.94</td><td>0.0036</td><td>-7.72</td><td>0.0031</td><td>-7.87</td></tr>
<tr><td>Charge transfer</td><td>0.8311</td><td>-2.02</td><td rowspan="2">0.2693</td><td rowspan="2">-2.67</td><td>0.0311</td><td>-3.828</td><td>0.0249</td><td>-3.92</td><td>0.0213</td><td>-3.97</td></tr>
<tr><td>Mass transfer limitation</td><td>0.4881</td><td>-1.28</td><td>0.0771</td><td>-3.18</td><td>0.0255</td><td>-3.45</td><td>0.0068</td><td>-3.52</td></tr>
</table>

Table S2. *The effect of KOH concentration on the resistance and time constant of the electrochemical phenomena occurring in the AEMWE cell at 1.8V in temperature of 50°C.*

**Potential**	**1.0M**		**0.5M**		**0.1M**		**Pure water**	
**Peak**	**R [Ω]**	$log(\tau)$	**R [Ω]**	$log(\tau)$	**R [Ω]**	$log(\tau)$	**R [Ω]**	$log(\tau)$
**Ohmic resistance**	0.0446		0.0552		0.0927		0.4790	
**Ionic transport**	0.0038	-6.94	0.0043	-6.68	0.0487	-4.18	2.1789	-3.43
**Charge transfer**	0.0311	-3.82	0.0733	-3.68	0.1324	-3.41	1.2070	-2.41
**Mass transfer limitation**	0.0771	-3.18	0.0595	-3.07	0.1661	-2.93	1.0638	-1.55

Table S3. *The effect of operating temperature on the resistances and time constants of the electrochemical phenomena occurring in the AEMWE cell at 1.8 V.*

**Potential**	**50 °C**		**60 °C**		**70 °C**		**80 °C**	
**Peak**	**R [Ω]**	**log** (τ)	**R [Ω]**	**log** (τ)	**R [Ω]**	**log** (τ)	**R [Ω]**	**log** (τ)
**Ohmic resistance**	0.0446		0.0427		0.0410		0.0398	
**Ionic transport**	0.0038	-6.94	0.0026	-7.45	0.0033	-7.57	0.0037	-7.24
**Charge transfer**	0.0311	-3.82	0.0323	-3.86	0.0299	-3.88	0.0289	-3.88
**Mass transfer limitation**	0.0771	-3.18	0.0678	-3.25	0.0665	-3.28	0.0595	-3.32

Table S4. *The effect of operating modes on the resistances and time constants of the electrochemical phenomena occurring in the AEMWE cell at 1.8 V and temperature of 80 °C.*

**Mode**	**Anode: 1.0 M KOH || Cathode: dry**		**Anode: Pure Water || Cathode: dry**		**Anode: 1.0 wt.% K_2CO_3 || Cathode: dry**		**Anode: 1.0 M KOH|| Cathode: 1.0 M KOH**	
**Peak**	**R [Ω]**	**log (τ)**	**R [Ω]**	**log (τ)**	**R [Ω]**	**log (τ)**	**R [Ω]**	**log (τ)**
**Ohmic resistance**	0.0373		0.6901		0.1486		0.0398	
**Ionic transport**	0.0000		2.2556	-3.81	0.2715	-3.50	0.0037	-7.24
**Charge transfer**	0.0252	-3.73	2.0127	-2.30	0.3689	-2.61	0.0289	-3.88
**Mass transfer limitation**	0.0581	-3.28	0.7804	-1.52	0.1321	-1.63	0.0595	-3.32

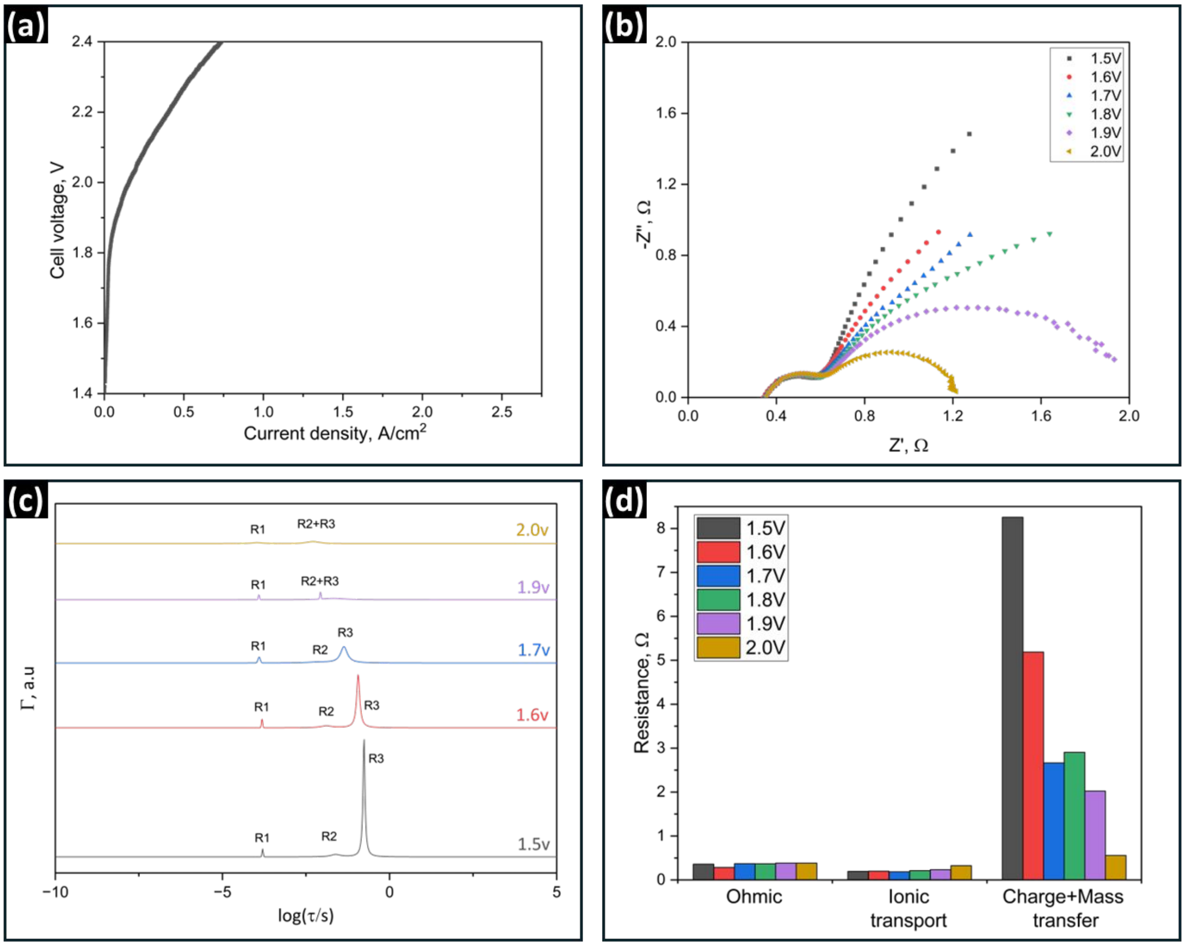

Figure S2. *(a) The polarization curve of an AEMWE cell utilizing AEMION AEM operated at 50°C with 1 M KOH as both anolyte and catholyte and feed flow rates of 5 mL/min. (b) Nyquist plots, (c) Normalized DFRT plots, and (d) Corresponding calculated effective resistance values of each peak at the different cell voltages ranging between 1.5-2.0 V.*
